# Supplementary material for: SNX19 Interacts with Caveolin-1 and Flotillin-1 to Regulate D1R Endocytosis and Signaling
Source: Biomedicines. 2025 Feb 15;13(2):481. doi: 10.3390/biomedicines13020481 (PMC11853350; doi:10.3390/biomedicines13020481)
Supplement: Supplementary file 1 [file biomedicines-13-00481-s001.zip › biomedicines-3339208-supplementary.pdf]

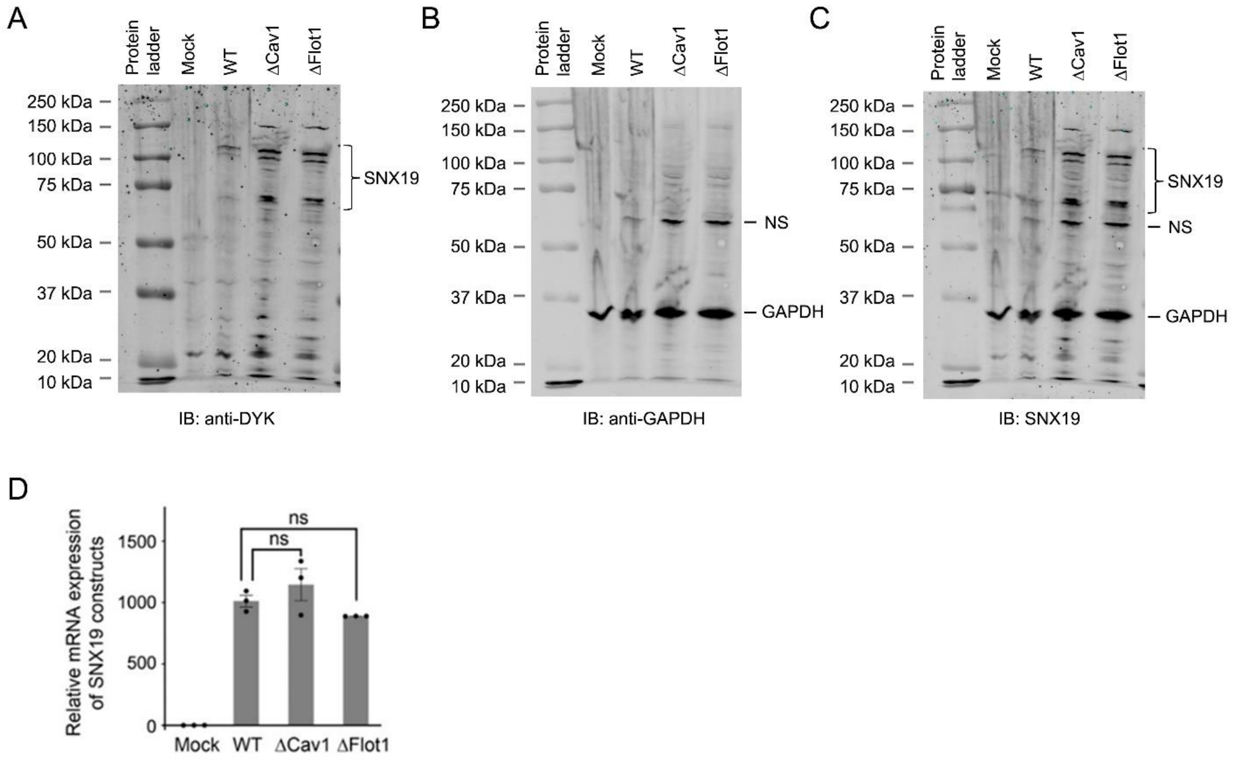

Supplementary Figure S1. Overexpression of WT-, ΔCav1-, and ΔFlot1-SNX19 in mouse RPTCs. (**A**) Plasmids of WT-, ΔCav1-, and ΔFlot1-SNX19 were transfected into mouse RPTCs as described in Methods. The cell lysates were subjected to SDS-PAGE and transferred onto nitrocellulose membranes, sequentially followed by immunoblotting with anti-DYK (**A**), anti-GAPDH (**B**), and anti-SNX19 (C) antibodies. The anti-DYK tag antibody is used to detect the overexpressed SNX19 protein; the anti-GAPDH is used for loading control; and the anti-SNX19 antibody is used to detect the endogenous SNX19. The expected molecular size of SNX19 is 109 kDa, which is observed from 70 kDa to 130 kDa. (**D**) Total RNAs were extracted with Trizol from cells that were transfected as in (**A-C**), using the procedures described in the Methods section. The relative mRNA expressions of wild-type (WT) and truncated SNX19 constructs were calculated using the average of individual values divided by γ-actin or Rplp0. N=3/group, ns: no significance. One-way ANOVA, Newman-Keuls test.

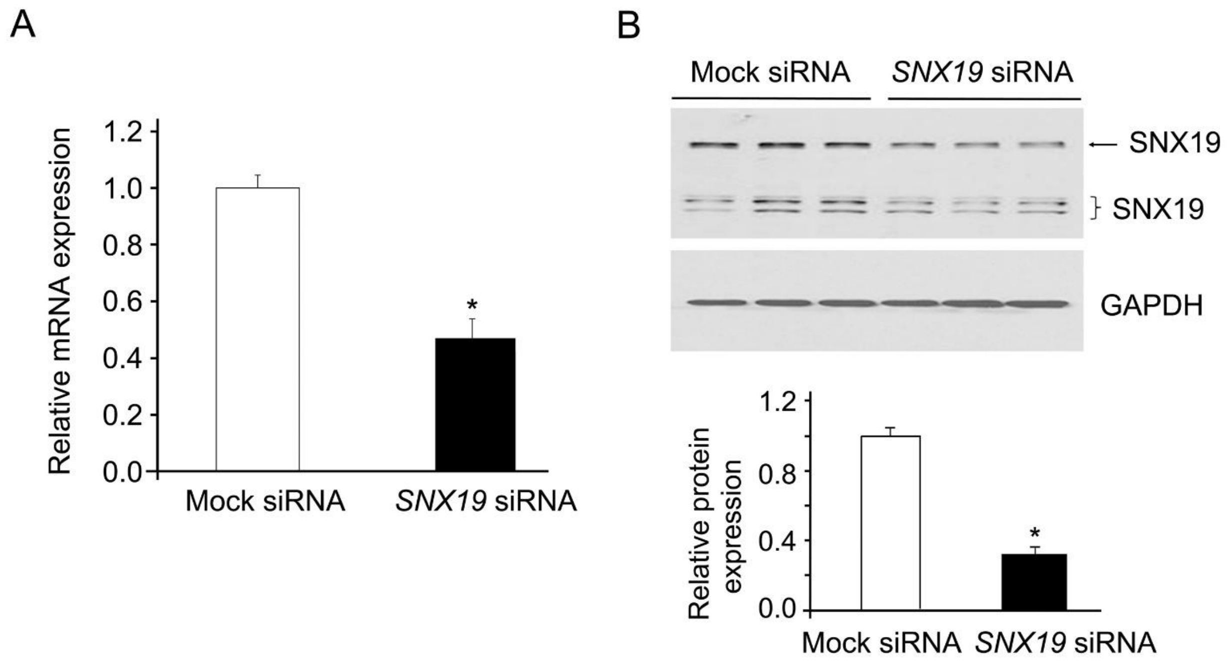

Supplementary Figure S2. Deficiency of SNX19 mRNA and protein expression in mouse RPTCs. Mock or specific *SNX19* siRNA were co-transfected with D_1R-GFP plasmid into human RPTCs as described in Methods. The cell pellets were harvested and divided into two portions. One portion is used for extraction of total RNA, another portion is used to generate cell lysates. (**A**) Total RNAs were extracted with Trizol from one portion of cells pellets, using the procedures described in the Methods section. The relative *SNX19* mRNA expressions of mock and specific *SNX19* siRNA transfection are shown. * P <0.05, n=4/group, Student's t test. (**B**) Cell lysates from another portion of cell pellets were run SDS-PAGE, nitrocellulose membranes were immunoblotted with anti-SNX19 antibody. Densities of bands indicated were quantified. * P <0.05, n=3/group, Student's t test.

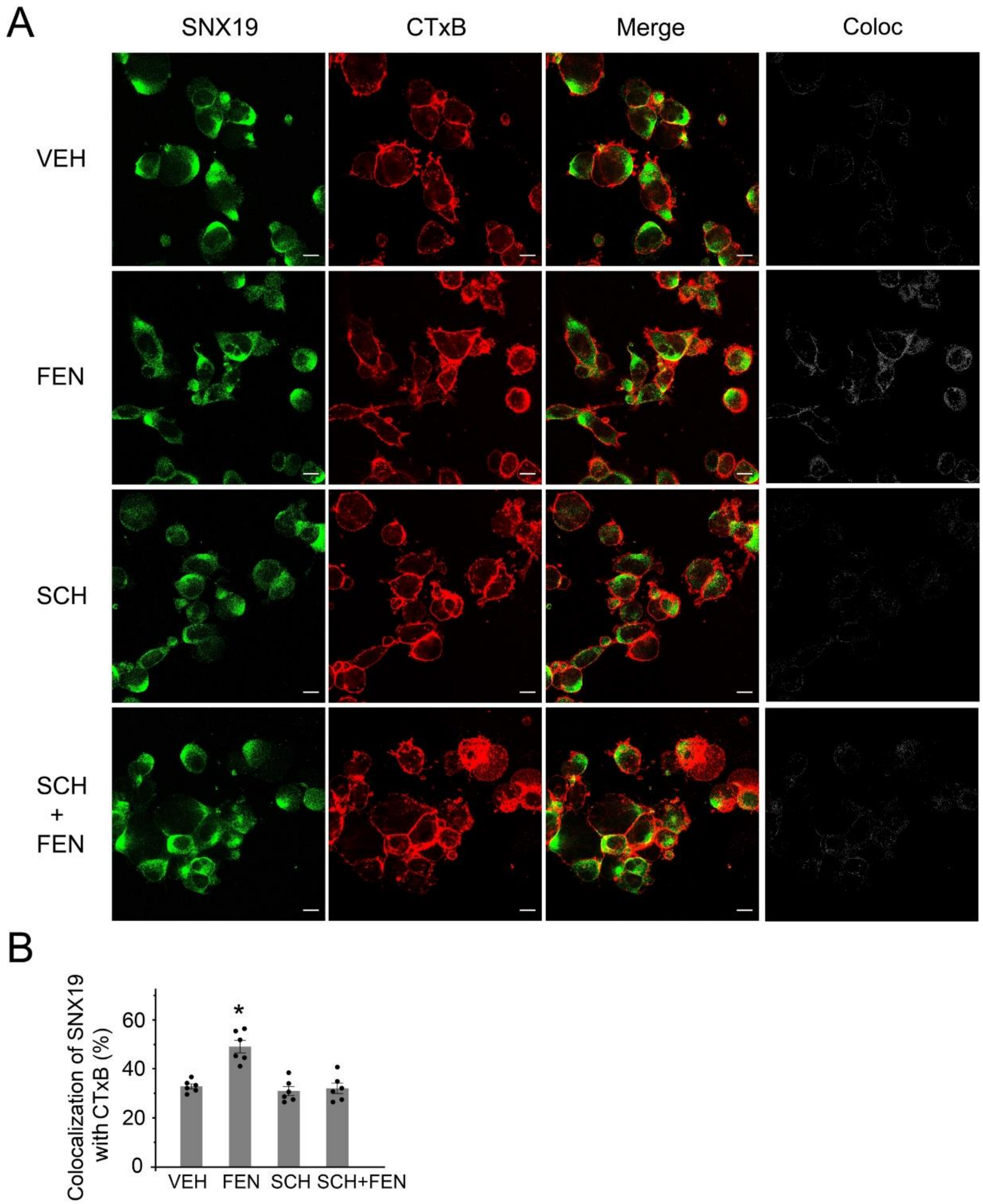

Supplementary Figure S3. D_1R-mediated regulation of SNX19 residence in LRs in mouse RPTCs. (**A**) Mouse RPTCs were treated with vehicle (VEH) or fenoldopam (FEN, 25 nM) in the absence or presence of SCH39166 (SCH), a D_1-like receptor antagonist, for 30 min. The RPTCs were fixed with 4% paraformaldehyde and stained with anti-DYK (for SNX19, green) and

cholera toxin B-subunit (CTxB, red); yellow in the merged images shows the co-localization of SNX19 with CTxB. Bar, 10 µm. A panel of separate colocalization images in “Coloc” were generated as described in Methods section. Coloc = colocalization. (**B**) Quantification of the co-localization of SNX19 with CTxB in the images from confocal microscopy. N=6, **P*<0.05 vs VEH, one-way ANOVA, Newman-Keuls test.

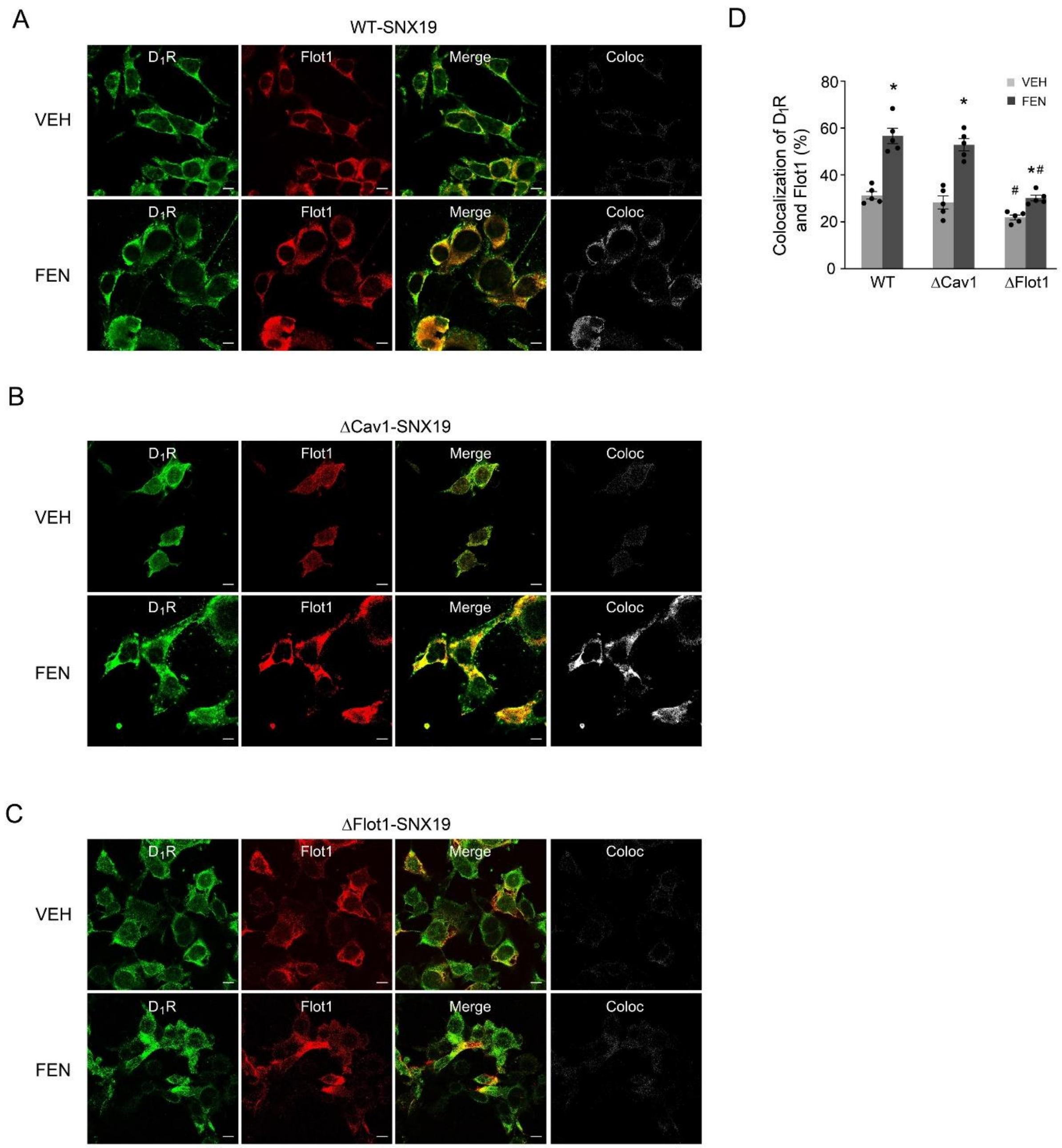

Supplementary Figure S4. Flotillin1-mediated D_1R endocytosis in WT-, ΔCav1-, and ΔFlot1-SNX19-transfected mouse RPTCs. (**A**) Mouse RPTCs, transfected with wild-type (WT) SNX19 plasmid, were treated with vehicle (VEH) or fenoldopam (FEN, 25 nM) for 30 min. The RPTCs were stained with anti-D_1R (green) and anti-Flot1 (red) antibodies. Bar, 10 µm. (**B**) Mouse RPTCs, transfected with SNX19 plasmid-deleted caveolin-1 binding motif (ΔCav1-

SNX19), were treated and stained as in (**A**). Bar, 10 µm. (**C**) Mouse RPTCs, transfected with SNX19 plasmid-deleted flotillin-1 binding motif (ΔFlot1-SNX19), were treated and stained as in (**A**) and (**B**). Bar, 10 µm. A panel of separate colocalization images in "Coloc" in (**A**)-(**C**) were generated as described in Methods section. Coloc = colocalization. (**D**) Quantification of the co-localization of D_1R with flotillin-1 (Flot1) in the images from confocal microscopy (**A-C**), N=5, *P<0.05 vs VEH, $^{\#}P$<0.05 vs WT, two-way ANOVA, Newman-Keuls test.

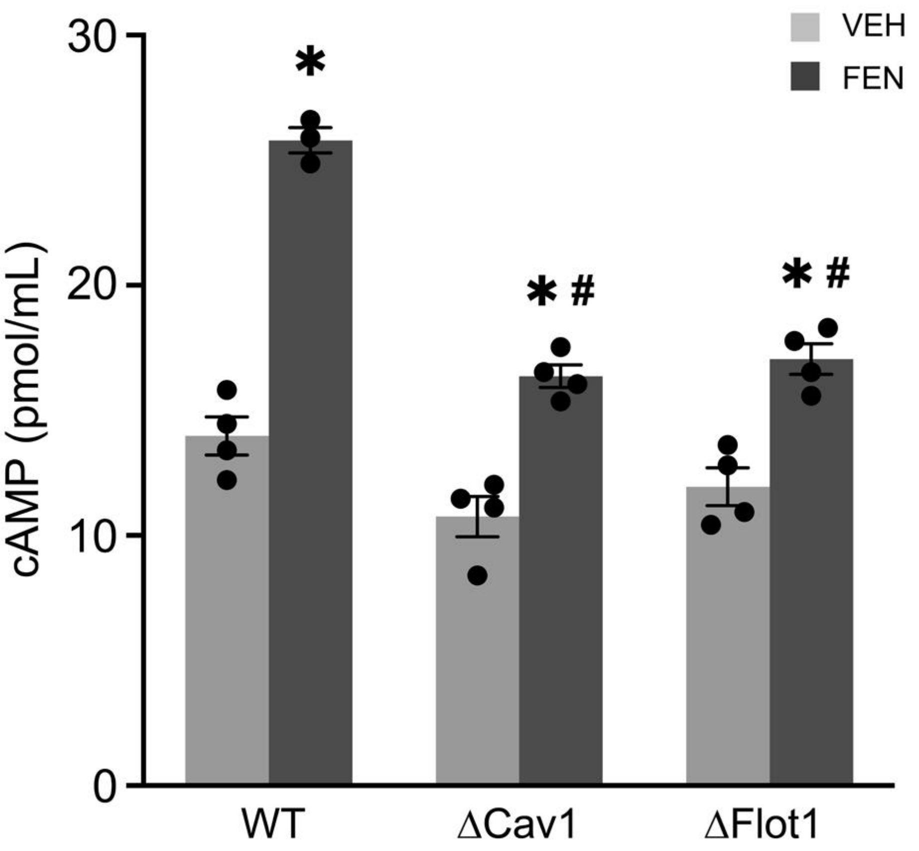

Supplementary Figure S5. Attenuation of cAMP production by deletion of either caveolin-1 or flotillin-1 binding motifs within SNX19. Mouse RPTCs transfected with WT-, ΔCav1-, or ΔFlot1-SNX19 plasmids were treated vehicle (VEH) or fenoldopam (FEN, 25 nM, 30 min). The cAMP concentrations in the supernatant were quantified by ELISA. N=4, **P* < 0.05 vs VEH, #*P* < 0.05 vs WT, one-way ANOVA, Newman-Keuls test.

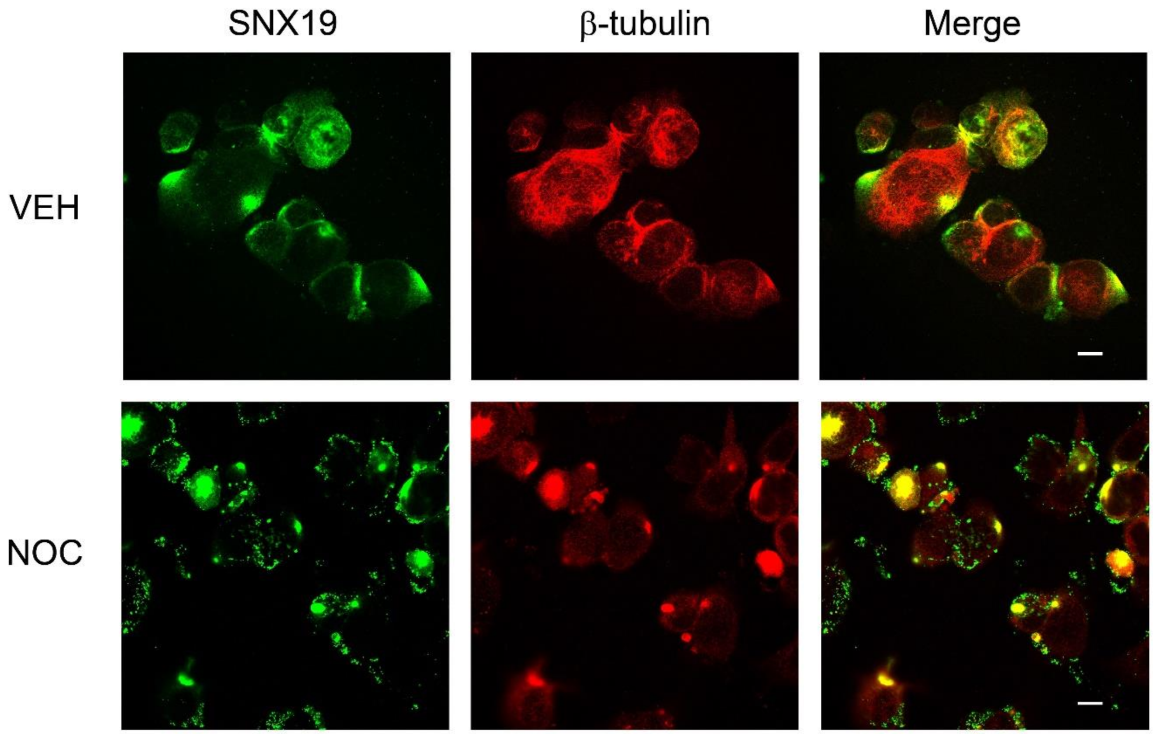

Supplementary Figure S6. Effect of microtubule polymerization inhibition on SNX19 and β-tubulin intracellular distribution. Mouse RPTCs transfected with wild-type SNX19 plasmid, were treated with vehicle (VEH) or nocodazole (NOC, 10 μM, 1hr), a microtubule polymerization inhibitor. The RPTCs were stained with anti-DYK (green, for SNX19) and anti-β-tubulin (red) antibodies. Bar, 10 μm.

Supplementary Table S1. Primers for RT-PCR for wild-type (WT-), and deleted caveolin-1 (ΔCav1-) or flotillin-1 (ΔCav1-) binding motif SNX19 constructs transfected in mouse RPTCs

Gene	Accession ID	Primer names	Primer sequences
ACTG1	NM_009609.3	γ-actin_foward<br>γ-actin_reverse	5’- CCCCCTGAACCCCAAAGCTAA -3’<br>5’- ATGACAATGCCAGTGGTGCG -3’
RPLP0	NM_007475	Rplp0_forward<br>Rplp0_reverse	5'- GCAGGTGTTTGACAACGGCA-3’<br>5'- CACAGACAATGCCAGGACGC-3’
N.A.	N.A.	pConstruct_forward<br>pConstruct_reverse	5'- TGTACCTGGGTGCCCCTTAG -3’<br>5'- CAGAATCCACCAAGCCCGTC -3’

N.A. = not applicable
